# Our National Disease

**Published:** 1876-01

**Authors:** P. H. Hayes


### OUR NATIONAL DISEASE.

P. H. HAYES, M. D.

It was a few years before the war of the Rebellion, that professional relations brought me into the family of Dr. Glassell, of Bowling Green, a place of considerable importance about forty miles this side of the city of Richmond, t[illegible] Capital of Virginia. The Doctor was the proprietor of a very large estate of nearly seventeen h[illegible]ndred acres of very rich land, lying upon the Matapony river, and he owned a sufficient number of slaves ("hands," he called them) to cultivate the larger part of this vast domain. In the course of my visit, I was shown over a part of this estate and through the "quarters" of the "hands." After this, the Doctor invited me to go to Richmond with him and spend a day in sight-seeing. We visited the slave auction-rooms, the great flouring mills, the fine bridge over the James, and finally the great tobacco manufacturing establishments, where a very large number of slaves—"hands"—were employed in putting up the leaf in all the various forms in which it meets our eyes in the markets. While we were looking on, the Doctor remarked that these hands were hired out by their owners in the country, to work the winter at this business, as there was little or no farm labor in the winter to be done on the estates But he instantly said: "I never hire a hand of mine to come and work in these close and warm rooms, through the winter; they get weak and sickly—spoils them for field hands; they're good for nothing but to lie around afterwards." The Doctor's sagacity in economy struck me forcibly. It was actually better for him in the long run that his hands should hunt and fish and lounge in their cabins, with fireplaces six feet wide and a door always open—be idle, in short, and live in the out-door air—than it was to let them work at high wages in a bad air through the winter.

I instantly thought of our six months of air-tight stores at the north; our double windows and double doors, and not, in one instance in a hundred, any constant or systematic provision for ventilation. I could not fail to think of the great numbers I had seen who had, in this way, become so tender and sickly that they had become afraid of fresh air —air cool and pure,—they verily thought it was their enemy and were always nervous about "too much air" in the room.

These people could hardly be made to believe, I suppose, that just the opposite habit can be formed, a wise dread of impure air. By accident, in our late war, a large hospital in a northern city was over-crowded, and the physician finally ordered a large number under tents in the spacious yard adjoining. In a short time there was room in the hospital, but nothing could induce the fever-stricken patients to go back, they had so rapidly improved in the pure, out-door air. I have heard the surveyors of wild lands—under the sky by day and a tent by night—say that it made them sick—the getting seasoned again to the atmosphere of houses.

There seems to be a sort of epidemic fascination for poisoned air, and doctors have often gained great credit by showing up, on a large scale, that they have for once, at least, understood the difference. A few years ago, an epidemic brought into Bellevue Hospital, in the city of New York, a hundred cases of typhus fever. The doctor in charge made every possible effort to save his patients, according to the books, and himself says: "We gave brandy to hold up the pulse, following the directions of Prof. Stokes, to begin when the first sound of the heart is almost gone; and so we did, and we went on trying to hold up the pulse, until seventy ounces of brandy was given to a single patient in a single day. But we found, after the fever had run a certain time, that we could not control the pulse in this way, and we stood by and gave the brandy on principle." Now, just at this time, and when *one* in *five* of these patients were dying, the doctor ordered every patient removed to the "Island" and placed under tents. And, he says, I ordered that not a drop of stimulants should go into those tents. I afterwards heard him declare in a public lecture, that here, with *fresh air* and *without stimulants*, he got the *best record ever shown* in typhus: only *one* in *sixteen* of these patients died. Dr. Hamilton "stamped out" an epidemic of cholera at the workhouse on Blackwell's Island, where there were eight hundred inmates, and already a hundred and twenty deaths, and this he did by taking every inmate *out of doors* for the day, and building fires in all the wards day and night, with windows and doors open. In *six days* the epidemic was at an end.

Such illustrations as I have given ought to awaken us all from our stupidity on the breath-oflife question. *Exhaustion* is our National Disease, and of all the physical influences at work in its causation, not one is everywhere so prevalent and so potent, as the breathing of bad air. [With the gradual banishment of the open fire-place, and the as gradual invasion of the great epidemic of air-tight stoves, there has come such an entire change in the type of diseases, as completely to have revolutionized the medical practice of forty years ago. Now all diseases, even acute ones, call for a supporting plan; *tonics* of some sort almost from the start. This general type of disease—the rapid tendency to death from exhaustion—is itself an index to the general low type of health among the masses. There is now almost no *robust* health to be seen, and for that very reason, no robust diseases which for a moment tolerate the heroic bleedings and mercurial practice of a generation ago. In our intense occupation with the present, the enterprises of our own time and the economies of wealth, we forget to study history for the lessons in human life. In the only authentic records of the first seventy-five hundred years of the world's history—the book of Genesis—the word sick does not occur but once, and so unknown a thing that a son should die before his father, that a record is made, a special record of one single instance of the kind: "And Haran died before his father Terah in the land of his nativity, in Ur of the Chaldees."

These ancient nations lived in the open air, slept in tents or upon house tops. They had no doctors, no hospitals, no asylums. They needed none.—Life was prolonged to hundreds of years, and death came as a real debt to nature, and not to the devil in the form of an air-tight stove.

I have said *the* disease of our nation is *exhaustion*. No matter how violent, apparently, the access of any disease, lowering treatment is not called for nor borne. If, under the influence of old education the practitioner begins this plan, he is almost certain to see his patient sink as soon as the disease begins to "turn," as the significant expression has it.

The plain inference from my argument is, that the majority of our nation are only half alive.—Don't forget where I began, that a Virginia planter had learned by experience and observation that it was better for him to lose the entire winter's wages of a gang of slaves and have them to board in the bargain, than that they should spend a winter in the warm and close rooms of a tobacco manufacturing establishment, and this, too, in a city so far south that really their winters are scarcely more than half as long as our own. Corresponding with what I have said, we find after our long winter of confinement in-doors is passing, and spring begins, then comes our sickly season. The tone of the system a little let down by the first warm weather, the debility of long confinement becomes apparent, and colds, coughs, catarrhs, rheumatism and neuralgia, pneumonia and scarlet fever and diphtheria are abundantly prevalent. We think we live in an age of great progress. I sometimes seriously doubt it. What does it mean, that in a single beautiful inland city in the famous State of New York, of about twenty thousand inhabitants, there are *fifty* physicians and *seventeen* drug stores! an average of one doctor to every four hundred inhabitants, and almost a drug store to every one thousand! while in the whole United States there is an average of one physician to every five hundred of our people. And such is the multiplication, refinement and complication of our maladies, that a higher standard of medical education is continually called for to meet the exigencies of our age.

If we are right in placing the beginning of our general physical degeneracy in the breathing of a vitiated and over-heated atmosphere, then it is a most interesting thing to observe how the violation of one great need of our system brings with it a tendency to violate every other. Thus the man poisoned and pale by impure respiration soon becomes a dyspeptic, and as soon as dyspepsia begins, stimulants of some sort or kind are craved and will be had, and the disease of the stomach and the recoil of stimulation creates palpitation, produces cerebral congestion, gradually destroys all power of concentration and consecutive thinking, closes the reason, perverts feeling, disenchants existence, and often shakes the foundations of character. It is intensely interesting thus to study the fact that the breaking of one great natural law helps to break every other, nay, makes it almost necessary to do so, and to observe also how the moment we keep inviolable a single great natural principle of health, how easy it is to keep all others.

